# Investigation of the Factors Related to Mortality and Length of Hospitalization among COVID-19 Patients in East Azerbaijan Hospitals, Iran

**DOI:** 10.34172/jrhs.2022.92

**Published:** 2022-10-11

**Authors:** Ali Abdi Tazeh, Asghar Mohammadpoorasl, Parvin Sarbakhsh, Madineh Abbasi, Abbasali Dorosti, Simin Khayatzadeh, Hossein Akbari

**Affiliations:** ^1^Department of Statistics and Epidemiology, Faculty of Health, Tabriz University of Medical Sciences, Tabriz, Iran; ^2^Tropical Diseases Research Center, Tabriz University of Medical Sciences, Tabriz, Iran; ^3^Department of Anesthesiology, Tabriz University of Medical Sciences, Tabriz, Iran; ^4^East Azerbaijan Province Health Center, Tabriz University of Medical Sciences, Tabriz, Iran

**Keywords:** Comorbidities, COVID-19, Inpatients, Length of stay, Mortality

## Abstract

**Background:** It is of utmost importance to identify populations with an elevated risk for COVID-19 and the factors influencing its outcomes. The present study aimed to investigate factors affecting mortality and length of stay (LOS) among COVID-19 patients in the hospitals of East Azerbaijan province, Iran, during 15 months of this pandemic.

**Study Design:** The present study followed a retrospective cohort design.

**Methods:** This retrospective study was conducted using data in the integrated syndromic surveillance system (ISSS) on patients admitted to the hospitals from February 21, 2020, to April 11, 2021. The association between variables of interest and death, as well as LOS, was investigated via multiple logistic regression and multiple linear regression analyses.

**Results:** In total, 24 293 inpatients with a mean age of 54.0 ± 19.4 years were included in this study. About 15% of them lost their lives, whose mean age was 69.0 ± 14.6 years, significantly higher than the recovered ones (*P* < 0.001). Factors, such as above 49 years of age (*P* < 0.001), male gender (OR = 1.17; 95% CI: 1.08-1.26), and having chronic diseases (OR = 1.32; 95% CI: 1.22-1.42), were correlated with patient mortality. In addition, having chronic diseases (Beta = 0.06; 95% CI: 0.03-0.08) was associated with higher LOS in hospitals.

**Conclusion:** In conclusion, older patients were at a higher risk of mortality and prolonged hospitalization. Furthermore, patients’ underlying diseases could cause a severe form of COVID-19, which can lead to death and increase patients’ LOS.

## Background

 Most COVID-19 cases have mild to moderate symptoms^1^; however, approximately 23% of them have severe diseases, and thus, the mortality rate is 5.6%^2^among them. Factors affecting the severity of this condition and the resulting mortality include male gender, ≥ 50 years of age, kidney disease, cerebrovascular disease, cardiovascular disease, respiratory disease, diabetes, hypertension, cancer, and obesity.^3-5^

 One significant aspect of epidemiological studies is to investigate the relationship between patients’ demographic characteristics, clinical signs and symptoms, and several underlying diseases, by which the severity of the disease and its outcomes, which could vary from patient to patient, can be determined, and then, to find the effects of these characteristics on disease outcomes. Moreover, knowing the temporal and spatial trends of the disease and its epidemiological aspects in different regions is one of the necessities for legislative decision-making to control and manage it. Therefore, using large population-based data, this study, as the first study in East Azerbaijan province, Iran, aimed to document the COVID-19 pandemic and investigate factors affecting mortality and length of stay (LOS) among patients in the hospitals of this province, during 15 months of this pandemic.

## Methods

 This descriptive-analytical study was conducted on COVID-19 patients admitted to the hospitals affiliated with Tabriz University of Medical Sciences (TBZMED), East Azerbaijan Province, Iran, from February 21, 2020, to April 11, 2021. In total, 24,293 patients who tested positive for COVID-19 by reverse transcriptase-polymerase chain reaction (RT-PCR) and were hospitalized in TBZMED hospitals were recruited for the study.

 The data were obtained from the integrated syndromic surveillance system (ISSS) of the Vice Chancellor’s Office for Health at TBZMED. The ISSS is a national electronic platform under the supervision of the Center for Communicable Disease Management of the Ministry of Health and Medical Education of Iran, which collects data on target syndromes from public and private healthcare providers. The main purpose of the ISSS is to quickly detect outbreaks of infectious diseases, particularly emerging ones, such as COVID-19. Data on COVID-19 suspected patients have been recorded as Severe Acute Respiratory Infections syndrome. All suspected cases were subjected to COVID-19 RT-PCR testing and confirmation. Incomplete data and patients without RT-PCR test results were excluded.

 Demographic characteristics, clinical signs and symptoms, COVID-19 RT-PCR test results, underlying diseases (diabetes, chronic respiratory/kidney/liver diseases, cancer, immune system deficiency, hypertension, chronic heart diseases, stroke, as well as other diseases), and disease outcomes (i.e., died or recovered) were retrieved from the ISSS. Additionally, data quality was evaluated for non-duplication and completeness.

 Descriptive statistics were used to describe the data. Chi-square test, *t* test, and analysis of variance (ANOVA) were utilized in univariate analysis. In addition, multiple logistic regression and multiple linear regression analyses were conducted in multivariate analysis. All statistical analyses were performed by the SPSS software (version 24).

## Results

 The mean age of patients was 54.0 ± 19.4 years. Totally, 12,603 cases (51.9%) were male with a mean age of 54.6 ± 19.3 years, and 11 690 cases (48.1%) were female with a mean age of 53.5 ± 19.4 years. Among all the hospitalized cases, 8,653 patients (35.6%) had at least one underlying disease.

 The most common complications of the disease among the inpatients were ground-glass opacity (n = 6532; 26.9%), bilateral pulmonary infiltration (n = 4350; 17.9%), and primary viral pneumonia (n = 2278; 9.4%).

 During the study period, 3663 patients (15.1%) lost their lives due to COVID-19. The mean age of the dead cases was 69.0 ± 14.6 years, among whom 2027 (55.3%) were male with a mean age of 68.7 ± 15.0 years, and 1636 were female with a mean age of 69.5 ± 14.11 years. The total case fatality rate (CFR) in this study was estimated to be 15.1% (minimum = 6.15% in April 2021 and maximum = 18.3% in December 2020). It was 16.1% for males and 14.0% for females (Table 1).

**Table 1:** Death rate and length of hospitalization by baseline characteristics of the hospitalized COVID-19 patients

**Variables**	**Died**		**Recovered**			**Length of hospitalization**		
	**N**	**%**	**N**	**%**	***P*** ** value**	**Mean**	**SD**	***P*** ** value**
Age group					0.001			0.001
< 18	17	3.7	438	96.3		4.68	7.1	
18-49	397	3.7	10 213	96.3		3.04	7.2	
50-64	876	10.0	4600	84.0		5.92	7.7	
65-79	1511	28.3	3823	71.7		6.72	8.5	
≥ 80	862	37.5	1434	62.5		6.50	7.4	
Total	3663	15.1	20 633	84.9		4.86	7.8	
Gender					0.001			0.001
Male	2027	16.1	10 579	83.9		5.15	8.1	
Female	1636	14.0	10 054	86.0		4.55	7.5	
Having underlying disease					0.001			0.001
Yes	2025	23.4	6628	76.6		6.33	7.6	
No	1638	10.5	14 005	89.5		4.05	7.8	

SD, standard deviation.

 The association between various factors and COVID-19 mortality was explored using multiple logistic regression analysis. As shown in Table 2, the odds of mortality due to COVID-19 increased with age, and the highest OR was over 80 for age. Additionally, the male gender and the underlying diseases significantly increased the chance of death due to COVID-19 in hospitalized patients.

**Table 2:** Result of multiple logistic regression analysis of factors associated with death and multiple linear regression for logarithm length of hospitalization

**Variables**	**Death**			**Length of hospitalization**		
**Age group**	**Adjusted OR**	**95% CI**	***P*** ** value**	**Beta**	**95% CI**	***P*** ** value**
< 18	Ref.			Ref.		
18-49	1.01	0.61-1.64	0.990	-0.05	-0.14-0.054	0.376
50-64	4.48	2.74-7.32	0.001	0.09	-0.01-0.19	0.082
65-79	9.06	5.55-14.79	0.001	0.09	-0.01-0.19	0.083
≥ 80	13.69	8.36-22.42	0.001	0.07	-0.04-0.167	0.235
Gender (male/female)	1.17	1.08-1.26	0.001	0.02	-0.03-0.05	0.080
Having underlying disease	1.32	1.22-1.42	0.001	0.06	0.03-0.08	0.001

 The median LOS was three days for patients (minimum = 0, maximum = 191 days). It was also three days for women (interquartile range [IQR] = 6) and three days for men (IQR = 7). Patients in the age group of 65-80 years experienced the highest LOS with a median of five days (IQR = 6). Moreover, the median LOS was five days in dead patients (IQR = 8) and three days (IQR = 6) for the recovered ones (Table 2).

## Discussion

 The present study aimed to investigate the epidemiological characteristics of COVID-19 and factors affecting mortality and LOS in COVID-19 patients admitted to hospitals affiliated with TBZMED, Tabriz, Iran. It was found that patients with older age, male gender, and underlying conditions had significantly higher mortality due to COVID-19. Additionally, the LOS was significantly higher for patients with older age and a positive history of underlying conditions.

 According to the results of the present study, CFR was 15.1% among hospitalized patients, and it was higher in men than women. Other studies have estimated the CFR of the COVID-19 disease in Iran as 13.5%^6^ and 8.1%.^7^ A systematic review that analyzed 39 studies calculated a CFR of 13%.^8^ In another systematic review involving 33 studies and 13,398 patients, CFR was estimated as 17.1%.^9^ These different fatality rates can be attributed to the ability of the healthcare system to respond to the pandemic, the predominant type of virus in the community at the time of the study, and the denominator of the different fractions in calculating the CFR of the disease.

 It was also found that higher age is the most influential factor in increasing the risk of COVID-19 mortality; therefore, the risk of mortality was the highest for patients above 80 years. Accordingly, 75% of all COVID-19 deaths were for those above 60 years. Numerous studies have further shown that old age is one of the essential predictors of patient mortality due to COVID-19.^10-13^ The high CFR in old age was also attributed to the point that most older people were suffering from several underlying diseases and were at a higher risk for death if they had become ill.^14^

 In this study, male patients experienced a higher risk of mortality due to COVID-19, and about 52% of deaths occurred in men. Several studies on COVID-19 patients indicated that males experienced higher mortality than females,^15-17^ which may be due to biological and socio-behavioral factors, as well as the difference in medication efficacy, comorbidities, immune system response, and sex-related hormonal differences.^17^

 Based on the findings of this study, higher age and underlying diseases were related to prolonged hospitalization. The median LOS in this study was three days for both male and female patients; however, patients in the age group of 65-80 years had the highest LOS at hospitals. Furthermore, the LOS in dead patients was about 1.6 times longer than that in the cases recovered and discharged from the hospitals. These results were similar to previous findings.^18-20^

 Considering that the ISSS data was used in this study, similar to other surveillance systems’ data, the present findings may have been affected by some errors in data collection, registration, and reporting, which cloud not be detected despite quality control efforts. Therefore, the results should be interpreted with caution.

**Highlights**

- Case fatality rate (CFR) was calculated to be 15.1% among COVID-19 hospitalized patients.
- The CFR is higher in male patients than the females.
- Aging and underlying diseases are major risk factors for prolonged hospitalization and death among COVID-19 patients.
- More than half of COVID-19 hospitalized patients have at least one underlying disease.

## Conclusion

 It was concluded that aging and underlying diseases were the most influential factors in COVID-19 mortality, as well as LOS at hospitals, among inpatient cases. More than half of the dead cases experienced at least one underlying disease, and the older ones had the highest LOS at hospitals. The CFR was higher in hospitalized men than women in all age groups. High-risk populations were also identified in terms of patient mortality and LOS at hospitals. The findings could be used in implementing the necessary prevention and treatment measures for high-risk groups to reduce COVID-19 mortality in hospitalized patients. Moreover, they could be utilized by health authorities to intensify their attempts in terms of educating high-risk groups to have more compliance with prevention measures and maintain the risk perception of the population at the desired level to voluntarily support healthy behaviors.

## Acknowledgments

 The authors hereby extend their gratitude to all healthcare workers contributing to this study, especially those involved in collecting patient information.

## Conflicts of interest

 The authors declare that they have no competing interests.

## Ethics approval

 This study was approved by the Ethics Committee of the Tabriz University of Medical Sciences, Tabriz, Iran, with the code of ethics as IR.TBZMED.REC.1400.007.

## Funding

 The study was supported by funding from the Centers for Tabriz University of Medical Sciences. The funding covered data collection, analysis, and report writing.
